# Bacteremia in a Newborn with Hypocalcemic Seizures and Vitamin D Deficiency

**DOI:** 10.1155/2021/9925707

**Published:** 2021-06-12

**Authors:** Rebecca L. Moore, Matthew L. Lorenz, Meghan E. Fredette, Lisa Swartz Topor

**Affiliations:** ^1^Department of Pediatrics, Brown University, Providence, RI, USA; ^2^Department of Internal Medicine, Rhode Island Hospital, Providence, RI, USA; ^3^Department of Internal Medicine, Brown University, Providence, RI, USA; ^4^Division of Pediatric Endocrinology, Hasbro Children's Hospital, Providence, RI, USA

## Abstract

Infants with neonatal hypocalcemia often present with seizures, and neonatal hypocalcemia can be due to parathyroid (PTH) insufficiency or resistance. Causes of hypocalcemia with PTH elevation include increased phosphate load, vitamin D deficiency (VDD) or defects in metabolism, renal dysfunction, hypomagnesemia, genetic mutations resulting in end-organ resistance to PTH, or critical illness. Hypocalcemia has also been shown to be associated with Gram-negative bacteremia and sepsis in adults. We present the case of a full-term, formula-fed newborn presenting with late-onset hypocalcemic seizures and VDD in the setting of *Klebsiella pneumoniae* bacteremia. This case highlights that newborns presenting with hypocalcemic seizures should undergo a workup for sepsis.

## 1. Background

Hypocalcemia in a term infant is defined as a total serum calcium <8 mg/dL or an ionized calcium <4.4 mg/dL. Late-onset hypocalcemia is defined as hypocalcemia that occurs in neonates after the second or third day of life. Most infants with hypocalcemia are asymptomatic; those who present with symptoms most commonly present with increased neuromuscular irritability or seizures [1] and less commonly with stridor, wheezing, or vomiting caused by laryngospasm, bronchospasm, or pylorospasm, respectively [2]. The majority of infants with hypocalcemia have parathyroid hormone (PTH) insufficiency, low vitamin D (25-hydroxy vitamin D), low magnesium, or are formula fed [3]. Treatment of symptomatic hypocalcemia in infants begins with administration of intravenous (IV) calcium gluconate followed by oral calcium for maintenance therapy and identification and treatment of contributing factors such as hypomagnesemia, hyperphosphatemia, and vitamin D deficiency (VDD) [4].

## 2. Case Report

A 17-day-old full-term formula-fed female infant was admitted to the hospital for seizures. Four days prior to presentation, parents noticed brief episodes of left-sided upper and lower extremity shaking and lip smacking with associated vomiting and decreased oral intake. She was not taking any medications or vitamins at home. Evaluation in the Emergency Department (ED) revealed an afebrile, nondysmorphic female infant with normal vital signs. Shortly after arrival, she had a seizure with upper and lower extremity shaking and increased drooling, which lasted for one minute and resolved without treatment. Initial labs showed total calcium 6.4 mg/dL (reference range 9–10.9), ionized calcium 2.8 mg/dL (4.2–5.2), magnesium 1.3 mEq/L (1.3–1.9), and phosphorus 9 mg/dL (3.4–5.9). Bicarbonate was 17 mEq/L (22–32) with an anion gap of 16 mEq/L. Lactic acid was 2.2 mEq/L (0.2–1.9) with a normal pH. Sodium, potassium, creatinine, liver function tests, complete blood count, urinalysis, and cerebral spinal fluid (CSF) studies were unremarkable. Urine, blood, and CSF cultures were collected. A noncontrast computed tomography scan of the brain was normal. Electrocardiogram (EKG) showed prolongation of the corrected QT interval (QTc) to 468 milliseconds. She received calcium gluconate (1 mL/kg of 10% calcium gluconate) and phenobarbital (20 mg/kg) intravenously. Repeat laboratory studies demonstrated low serum calcium at 6.8 mg/dL (9–10.9) and persistently high phosphorus of 7.9 mg/dL (3.4–5.9). Additional tests showed elevated PTH of 143 pg/mL (18–80), low vitamin D of 7.4 ng/mL (>30), and 1.25(OH)D of 56 pg/mL (31–87). Thyroid-stimulating hormone (TSH) was normal. Maternal labs revealed normocalcemic VDD with a vitamin D of 9.4 ng/mL.

The infant was admitted to the hospital and treated with oral calcium carbonate (100 mg/kg/day of elemental calcium), phenobarbital, and broad-spectrum antimicrobial therapy with acyclovir, ampicillin, and gentamicin.

**Figure 1:**
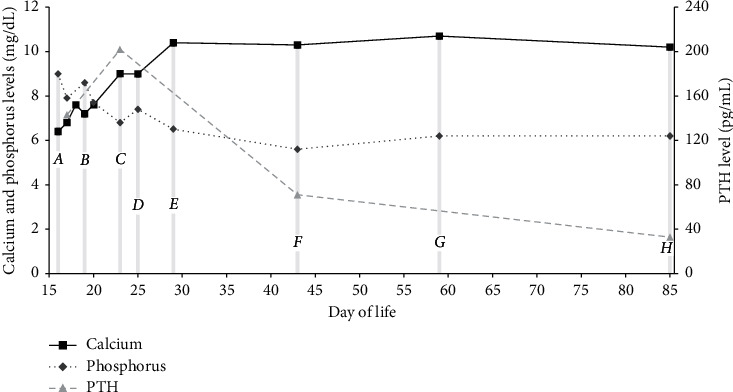
Initiated calcium carbonate at 100 mg/kg/day and low-phosphate formula (A: day of life (DOL) 16) and cholecalciferol at 2000 units/day (B: DOL 19). Weaned calcium carbonate to 75 mg/kg/day (C: DOL 23), 56 mg/kg/day (D: DOL 25), and 26 mg/kg/day (E: DOL 29). Transitioned to regular formula (F: DOL 43). Discontinued calcium carbonate (G: DOL 59). Weaned cholecalciferol to 400 units/day (H: DOL 85).

Blood cultures grew *Klebsiella pneumoniae*, and antimicrobial therapy was changed to cefazolin based on sensitivities. Urine and CSF cultures were negative; repeat blood cultures obtained on the third day of admission were negative. The patient started low-phosphate formula and cholecalciferol on the third day of admission. Serum calcium normalized by day 4 of calcium therapy. Magnetic resonance imaging of the brain, obtained due to concern for focal seizures on presentation, was normal. Repeat EKG showed normalized QTc. After discharge home, laboratory studies were closely monitored, and calcium supplementation and phenobarbital were gradually tapered. At seven weeks of age, she resumed cow's milk infant formula with continued normal calcium, phosphorus, and PTH levels (Figure 1).

## 3. Discussion

We describe an infant with late-onset hypocalcemia with multiple potential contributing factors including (1) VDD with biochemical findings resembling pseudohypoparathyroidism, (2) use of cow's milk formula, and (3) Gram-negative rod bacteremia. While the association between bacteremia and hypocalcemia has been described in adults, it has not been previously reported in infants or children. Thorough diagnostic evaluation allowed for recognition and treatment of both hypocalcemia and bacteremia.

In a retrospective review, Thomas et al. summarized clinical and laboratory characteristics as well as outcomes of neonates presenting with transient late-onset hypocalcemia. The majority of patients in this study had hypomagnesemia, low vitamin D, and PTH insufficiency with low or inappropriately normal phosphorus; none had bacteremia [1]. In contrast, our patient was found to have normal magnesium level, *Klebsiella* bacteremia, and VDD with elevated PTH and high serum phosphorous.

VDD is consistently reported in neonates with hypocalcemia [1, 3]. However, there are no well-established thresholds for vitamin D levels of clinical significance nor a clear relationship between low vitamin D and symptomatic hypocalcemia. Yilmaz et al. measured vitamin D, calcium, and PTH levels in 750 infants and found significantly lower vitamin D levels in preterm infants with hypocalcemia, but no significant difference in vitamin D levels between hypocalcemia and normocalcemia in term infants [5]. Nevertheless, there are multiple reports of hypocalcemic seizures in infancy attributed to VDD [6, 7]. A population-based study in the United Kingdom estimated the incidence of hypocalcemic seizures due to VDD to be 3.49 per million children 0–15 years of age (95% CI: 2.81–4.26) and 44.36 per million in children 0-1 year of age (95% CI: 35.12–55.28) [8]. The increased incidence of hypocalcemic seizures in infancy may be related to maternal VDD, which leads to inadequate passage of vitamin D across the placenta during pregnancy and predisposes the infant to hypocalcemia. Interestingly, phosphate levels vary in infants with VDD in contrast to older children with VDD, who classically have low-to-normal phosphorus levels due to secondary hyperparathyroidism [3, 6].

The term pseudohypoparathyroidism (PHP) encompasses a heterogeneous group of disorders characterized by end-organ resistance to PTH, which can be accompanied by features of Albright Hereditary Osteodystrophy (AHO) and resistance to other hormones [9]. The AHO phenotype includes short stature, subcutaneous ossifications, obesity, and brachydactyly, among other findings. Type 1 PHP has an impaired 3′,5′-cyclic adenosine monophosphate (cAMP) response to PTH infusion. Type II PHP is characterized by a normal cAMP response to PTH infusion, without a phosphaturic response, and has no AHO phenotype or other hormonal resistances. VDD has been described in previous reports to mimic PHP type II, with a clinical picture of PTH resistance, manifesting biochemically as hypocalcemia, elevated PTH and phosphorous, and low urinary phosphorus excretion despite typical cAMP generation [10]. The PTH resistance in these cases of VDD was responsive to vitamin D supplementation, with re-established phosphaturic response to PTH after vitamin D repletion. Additionally, neonates can have transient PTH resistance that has been proposed as a developmental condition of the kidney that resolves with time [11]. Our patient had a clinical picture resembling PHP type II, which was responsive to treatment with vitamin D, as has been previously reported.

Excess phosphate intake has also been reported as a cause of late-onset hypocalcemia, including in infants fed bovine-milk formula with a high phosphorus concentration [12] and in other forms of phosphate administration, such as with phosphate enema use. Elevated phosphorus results in calcium precipitation, with resultant hypocalcemia and increased PTH. Although excess phosphorus intake from standard infant formula has been described to cause hypocalcemia, it was unlikely the only contributing factor in this case given the use of standard infant formula with age-appropriate volumes.

*Klebsiella pneumoniae* bacteremia may have also contributed to our patient's hypocalcemia. A review of the adult literature reveals an association between hypocalcemia and sepsis from Gram-negative bacteria. In a study of 60 critically ill patients with bacterial sepsis by Zaloga and Chernow, only those with Gram-negative sepsis developed hypocalcemia, and calcium levels normalized in those patients who survived after treatment for infection [13]. Similarly, a study by Aderka et al. found that 37% of patients with bacteremia had hypocalcemia, compared with 4% of patients without bacteremia; however, the incidence and magnitude of hypocalcemia in Gram-positive and Gram-negative bacteremia were similar. Patients with normocalcemia and hypocalcemia both experienced significant hypoalbuminemia and impaired renal function compared to those without bacteremia [14]. Desai et al. found a strong association between sepsis and hypocalcemia with higher mortality rates in patients with hypocalcemia (44% mortality with hypocalcemia versus 17% without), suggesting calcium dysregulation may be an indicator of disease severity [15]. Although the mechanism for the association between Gram-negative bacteremia and hypocalcemia is not fully elucidated, animal studies have shown that administration of endotoxin induces hypocalcemia most likely by impairing calcium mobilization [16]. Multiple pediatric studies suggest an association between hypocalcemia and sepsis, particularly in neonates; however, these studies largely focused on critically ill children, for whom the etiology of hypocalcemia was often uncertain and potentially multifactorial. These reports did not identify any association between hypocalcemia and bacteremia in children with noncritical illness [17–19].

In addition, immune system activity involves vitamin D metabolism and receptor activation [20]. As such, VDD may increase susceptibility to infection, as suggested by associations of inadequate vitamin “*D*” with confirmed sepsis and worse outcomes compared to controls in an observational study of pediatric patients in an intensive-care unit [21]. VDD in our patient may have placed her at higher risk of developing bacteremia. Further studies are needed to better understand if there is a role for vitamin D supplementation in pediatric infections [22].

## 4. Conclusions

We describe an infant with late-onset transient neonatal hypocalcemia with VDD who was also found to have Gram-negative bacteremia. The association between hypocalcemia and Gram-negative bacteremia has been reported in adults but has not been described in children outside of the setting of critical illness. Our case suggests that there may be an association between infection and hypocalcemia in a susceptible infant. Based on our case and review of the literature, we suggest that infection be considered as a possible contributing factor in newborns presenting with hypocalcemic seizures.

## Abbreviations

AHO: Albright hereditary osteodystrophy
CSF: Cerebrospinal fluid
DOL: Day of life
ED: Emergency department
EKG: Electrocardiogram
IV: Intravenous
PHP: Pseudohypoparathyroidism
PTH: Parathyroid hormone
QTc: Corrected QT
TSH: Thyroid-stimulating hormone
VDD: Vitamin D deficiency.
